# Maternal and perinatal outcomes of hypertensive disorders of pregnancy in Ethiopia: systematic review and meta-analysis

**DOI:** 10.1186/s12884-019-2617-8

**Published:** 2019-12-03

**Authors:** Amanual Getnet Mersha, Tadesse Melaku Abegaz, Mohammed Assen Seid

**Affiliations:** 1 Department of Gynecology and Obstetrics, School of Medicine, College of Medicine and Health Sciences, University of Gondar, P.O. Box: 196, Gondar, Ethiopia; 2 Department of Clinical Pharmacy, School of Pharmacy, College of Medicine and Health Sciences, University of Gondar, P.O. Box: 196, Gondar, Ethiopia

**Keywords:** Ethiopia, Hypertensive disorders of pregnancy, Meta-analysis, Outcomes, Systematic review

## Abstract

**Background:**

Hypertensive disorders of pregnancy complicate around 6% of pregnancies and accounts for 19% of maternal death in Ethiopia. The current review aimed to assess maternal and perinatal outcomes of pregnancies complicated by hypertension in Ethiopia.

**Methods:**

A systematic review and meta-analysis was done on the outcome of hypertensive disorder among pregnant women in Ethiopia. Literature search was made in five databases and Statistical analyses were carried out by using Stata 14 software. The pooled prevalence of maternal death, HELLP syndrome, perinatal death, and low birth weight was calculated using a random-effects model. Egger’s test and funnel plot were used to evaluate publication bias. The Cochran Q test and I^2^ test statistics were used to test the heterogeneity of studies.

**Result:**

Thirteen studies included in the review, with an overall sample size of 5894 women diagnosed to have hypertensive disorder of pregnancy. The pooled prevalence of maternal death was estimated to be 4% (95% CI: 2, 6%). The pooled prevalence of HELLP syndrome was 13% (95% CI: 10, 16%). Other complications such as pulmonary edema, kidney injury, hepatic injury, placental abruption, and aspiration pneumonia were also reported. Perinatal death was observed in one-fourth of women with HDP 25% (95% CI: 18, 32%). The pooled prevalence of low birth weight neonate in a woman with HDP is 37% (95% CI, 27, 48%).

**Conclusions:**

In Ethiopia, the prevalence of perinatal and maternal mortality among pregnant women with one of the hypertensive disorders were found to be higher than rates reported from high income as well as most of the low and middle income countries. For instance, one in four of pregnancies complicated by hypertensive disorder end up in perinatal death in Ethiopia. HELLP syndrome, placental abruption, pulmonary edema, renal damage, prematurity, perinatal asphyxia, and low birth weight were also commonly reported. To improve the health outcomes of hypertensive disorders of pregnancy, it is recommended to improve utilization of maternal health service; early detection and early referral of pregnant women with hypertensive disorder; advocating policies and strategies that improves the quality of health care that a pregnant woman and her newborn receive.

## Background

Globally, hypertensive disorders of pregnancy complicate 3–10% of all pregnancies and it is a major cause of maternal and perinatal complications [1]. A recent review reported that hypertensive disorders of pregnancy complicate around 6% of all pregnancies in Ethiopia [2]. Hypertensive disorders of pregnancy (HDP) accounts for 18% of maternal deaths worldwide, with an estimated number of about 62, 000–77, 000 deaths occur each year [3]. Due to the existing low level of health service utilization and poor quality of maternal and neonatal care, the maternal and perinatal morbidities are much higher in low and middle -income countries (LMICs) [4, 5]. For instance, 19% of all maternal deaths in Ethiopia are attributed to hypertensive disorders of pregnancy [6]. Hypertensive disorders of pregnancy were also reported to account for 30% of maternal mortality in Ghana [5].

Maternal complications of hypertensive disorders of pregnancy include placental abruption, pulmonary edema, thrombocytopenia, hemolytic anemia, stroke, recurrent seizure, renal damage, hepatic injury and others [7]. HELLP syndrome comprises of the following: haemolysis, elevated liver enzymes, and low platelets. HELLP syndrome occurs in about 0.5 to 0.9% of all pregnancies and complicates 10 to 20% of women with severe preeclampsia. HELLP syndrome is one of the common cause of maternal and fetal mortality among pregnant women with hypertension [8].

According to the 2016 Demographic and Health Survey (EDHS), the perinatal mortality rate in Ethiopia was 33 per 1000 pregnancies by the end of 2016 [9]. Perinatal mortality is three to five folds higher in women with preeclampsia/eclampsia syndrome as compared to those without the disorders [10, 11]. Different studies documented high perinatal mortality rate among women with hypertensive disorders of pregnancy. For instance, the rate of perinatal death was found to be 317/1000 births in Ethiopia [29]; 230/1000 births in Pakistan [12]; and, 144/1000 births in Turkey [13]. A Southern Ethiopia study reported a considerable association of perinatal death with maternal death, antepartum occurrence of the disease, low birth weight, hepatic injury, earlier gestational age at diagnosis, having eclampsia, and multipara [22].

Although hypertensive disorders of pregnancy are among the leading causes of maternal and perinatal deaths in Ethiopia, there is no pooled national evidence that demonstrates the feto-maternal outcomes of pregnancies complicated by the disorder. Therefore, the current review aimed to assess the maternal as well as perinatal outcomes of pregnancies complicated by hypertension in Ethiopia.

## Methods and materials

### Study design and search strategy

A systemic review and meta-analysis was conducted to assess maternal and fetal outcomes of hypertensive disorders of pregnancy in Ethiopia. The studies were retrieved through internet search from the databases of MEDLINE, Scopus, PubMed, ScienceDirect, and Google Scholar. A combination of keywords and phrases like: preeclampsia (Mesh), preeclamp*(all fields), eclampsia (Mesh),eclamp*(all fields), hypertensive disorders of pregnancy (Mesh), hypertensive disorders of pregnancy (all fields), fetal outcome (Mesh), fetal outcome (all fields), maternal outcome (Mesh), maternal outcome (all fields), gestational hypertension (Mesh),gestational hypertension (all fields), pregnancy induced hypertension (all fields), and Ethiopia (all fields), were used to search articles in the databases. The reference lists of identified studies were also screened to recover other articles and one unpublished study was retrieved from Addis Ababa University electronic library. All published articles up to 21 September 2018 were included in the review.

### Eligibility criteria

All observational studies that reported at least one of the maternal and/or fetal outcomes of hypertensive disorders of pregnancy and conducted in Ethiopia were included in the current review.

### Operational definitions of outcomes

The primary outcomes of interest included in the Meta-analysis are maternal death, HELLP syndrome, perinatal death, and low birth weight. The secondary outcomes included in the review are preterm delivery, perinatal asphyxia, acute kidney injury, aspiration pneumonia, pulmonary edema, ANC service utilization, and placental abruption.

#### Maternal death

The death of a woman while pregnant or within forty-two completed days of termination of pregnancy irrespective of duration and site of pregnancy, from any cause related to or aggravated by the pregnancy or by its management but not due to accidental or incidental causes.

#### HELLP syndrome

A syndrome consists of haemolysis (H), elevated liver enzymes (EL), and Low platelet count (LP).

#### Perinatal death

The death of a fetus/neonate in the perinatal period (from age of viability or twenty-eight weeks of gestation in Ethiopian context to first six days after birth).

#### Preterm delivery

Birth of baby after age of viability or twenty-eight weeks of gestation in Ethiopian context but before thirty-seven completed weeks of gestation.

#### Low birth weight

Birth weight of less than 2500 g.

#### Perinatal asphyxia

A neonatal condition defied by five minute APGAR score of less than seven.

### Data extraction

All of the research articles that were identified from searches of the electronic databases were imported into the ENDNOTE software version X5 (Tomson Reuters, USA) and duplicates were removed. Two authors (AGM and TMA) screened the titles and abstracts of identified articles by applying the inclusion criteria. Two authors (AGM and MAS) independently reviewed the full text. Final inclusion of the studies was determined by agreement of both reviewers and when there is disagreement, a third author (TMA) was involved. All the authors were involved in the discussion and agreed on the final inclusion. Before data extraction had begun, full-length articles of the selected studies were read to confirm for fulfilling the inclusion criteria. Then, data extraction was performed by two reviewers (AGM and TMA) independently. The selected studies were reviewed to extract data like; year of publication; author(s); study design; sample size; maternal outcomes; fetal outcomes; type of HDP; period of occurrence of the HDP; gestational age at the time of diagnosis; and antenatal care visit. When there was a disagreement in data extraction between the reviewers, it was resolved through discussion and mutual agreement between the investigators.

### Quality assessment

All reviewers (AGM, MAS, TMA) independently assessed the quality of studies using strengthening the reporting of observational studies in epidemiology (STROBE) scale checklist quality assessment tool [17]. All of the included studies were assessed to have a quality of > 70% and, there were no studies excluded based on quality assessment.

### Statistical analysis and heterogeneity

Statistical analyses were carried out by using Stata 14 (Stata Corp LP, College Station, TX) software to estimate the pooled prevalence of selected maternal and perinatal outcomes [18]. Statistical heterogeneity between studies was evaluated using the Cochran’s Q test and I^2^ statistic [19]. I^2^ value of greater than 75 demonstrates that heterogeneity among the studies is high and probably a few studies are contributing to the final result. Random-effects model for estimating pooled effects was employed due to the high level of observed heterogeneity and was measured as proportions of outcomes with 95% confidence intervals (CIs). Egger’s regression asymmetry test with *p-value* < 0.05 used as a cutoff to declare presence of statistically significant publication bias. The detail description of the original studies was presented in a table and forest plot.

## Results

### Studies identified

**Fig. 1:**
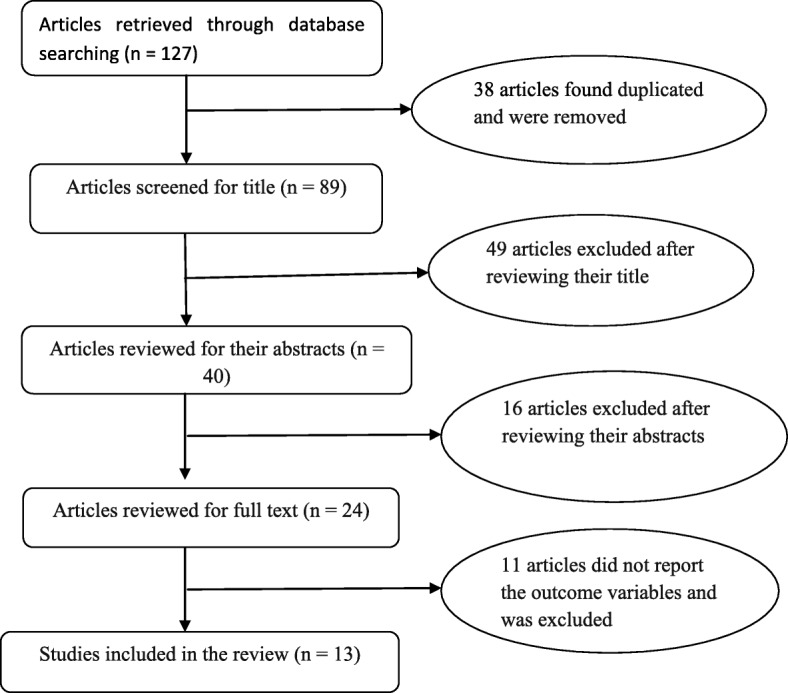
Flow diagram of the studies included in the review

A total of 127 articles were identified from five electronic databases (MEDLINE, Scopus, PubMed, ScienceDirect, and Google Scholar). Out of these identified articles: 38 articles found duplicated and were removed; 49 articles excluded after reviewing their title; 16 articles excluded after reviewing their abstracts; and 11 articles excluded after a full text review (did not report the outcome variables). Finally, thirteen studies were included in the systematic review and meta-analysis (Fig. 1).

### Description of the studies

Out of thirteen studies as demonstrated in table one; eleven studies were cross-sectional studies [20, 21, 23–26, 28–32] while two studies followed a retrospective cohort study design [22, 27]. Four studies conducted in Southern Nations Nationalities and People (SNNPR) [22, 23, 27, 30]; four studies were conducted in Addis Ababa (capital city of Ethiopia) [24, 25, 31, 32]; three studies were from Oromia regional state [20, 26, 29]; one study in Amhara region [28]; and one study conducted in Somali regional state of Ethiopia [21]. The sample size of studies ranges from a minimum of 93 women [21] to a maximum of 1809 women [25]. All of the included studies defined hypertensive disorders of pregnancy as a Systolic blood pressure (SBP) of 140 mmHg or higher and/or diastolic blood pressure (DBP) of 90 mmHg or higher on two or more consecutive occasions during pregnancy.

### Socio-demographic, ANC service utilization, and clinical characteristics of study participants

Overall, thirteen studies with a total sample size of 5894 pregnant women were found to have one of the HDPs and included in the review [20–32]. A maximum of 52.5% in a study conducted in Jimma specialized hospital [20] to a minimum of 3.5% among women admitted at teaching hospitals in Addis Ababa [24] were not having any antenatal care visit. Furthermore, 52% of women from another study [26]; 22.2% from a study in Amhara region [28]; and, 7.5% in Somalia regional state of Ethiopia [21] had no antenatal care visit. For other components of socio-demographic and clinical characteristics of women included in the review there were no adequate data to generate results.

### Hypertensive disorder of pregnancy (HDP)

Among the five groups of HDP, preeclampsia – eclampsia syndrome was the most commonly reported type of HDP. For instance, preeclampsia accounts for 66.2% of mothers among women admitted with HDP in Jimma specialized hospital [20]; 72.9% among mother with HDP in a Southern Ethiopian study [23]; and, 82.7% in a study done in Addis Ababa [25]. Eclampsia was reported to account for 27.8% of women admitted for HDP in a study conducted at Amhara regional state [28]; 34.1% in Southern study [27]; and, 24.2% in a study from Addis Ababa study. A study conducted in Somalia regional state of Ethiopia reported that 61% of the women with HDP were diagnosed during the antepartum period; 28%were diagnosed during the intrapartum period; and the remaining 10.7% were diagnosed in the postpartum period [21]. Most convulsions started during the antepartum period as compared to onsets during the intrapartum and postpartum periods. For instance, 38% of all convulsions in a study conducted at Oromia regional state started in the antepartum period; 18% occur during the intrapartum period; and the rest 44% convulsions started during the postpartum period [20]. Additionally, 86% of all the convulsions started during the antepartum period in a study conducted at teaching hospitals located at Addis Ababa [24] (Table 1).

**Table 1:** Overview of studies included in the systematic review and Meta-analysis

Sources	Location	Study design	Quality (%)	Sample size	No ANC	PND	SB	END	LBW	PTB	PNA	MD	Renal injury	Hepatic injury	HELLP Syndrome	Pulmonary edema	Placental Abruption	Preeclampsia	Eclampsia	Others
Wondimu et al. (2018)	Karamara hospital	Cross sectional	77.3%	93	59	26	19	7	34	47	26	10	22	3	5	9	7		93	
Obsa and Wolka (2018)	Wolita	Cross sectional	81.8%	225	34							4	16			13		164	58	3
Seid et al. (2017)	Gandhi Memorial hospital, Addis Abeba	Cross sectional	72.7%	200	7							1	13		28			200		
Maereg Wagnew et al. (2016)	Tikur Anbesa, Zewditu, St. Paul’s hospitals, Adis Abeba	Cross sectional	83.4%	1809	387	513	363	150	532	395	102	6			257	114	100	1496	313	
Nega et al. (2015)	Jimma university hospital	Cross sectional	82.1%	314	165							23	76		57	82		208	106	
Seyom et al. (2015)	Mettu Kari hospital	Cross sectional	72.9%	121	62	13	11	2	46	34	20		8	14	15		2	76	23	22
Yifruet al. (2015)	Southren Ethiopia	Retrospective cohort	71.2%	1015	382							51	521	326				612	346	57
Wubanchi et al. (2015)	Debre Berhan hospital	Cross sectional	81.2%	270	49	81			89	56	88	7						182	75	13
Vata et al. (2015)	Dilla University hospital	Cross sectional	75%	172		16	16		23		27						2	143	29	
Selamawit et al. (2015)	Zewditu Memorial hospital	Cross sectional	76.5%	250	14	29	19	10	126	91	132		20		38		15	121	17	112
Endesha et al. (2014)	Hawassa,Hosanna, Yirgalem Hospital	Retrospective cohort	77.9%	1015	382	322	261	61	545			51	521	326				612	346	57
Zenebe et al. (2011)	Jimma university hospital	Cross sectional	74.8%	153	40	52	42	10	70		33				14			108	37	8
Abate et al. (2006)	St. Paul’s and Tikur anbesa hospitals	Cross sectional	73.5%	257		113	69	44	25			28								

*PND* perinatal death, *SB* still birth, *END* early neonatal death, *LBW* ow birth weight, PNA perinatal asphyxia, PTB preterm birth, *MD* maternal death, ANC antenatal care

### Maternal outcomes of HDP

**Fig. 2:**
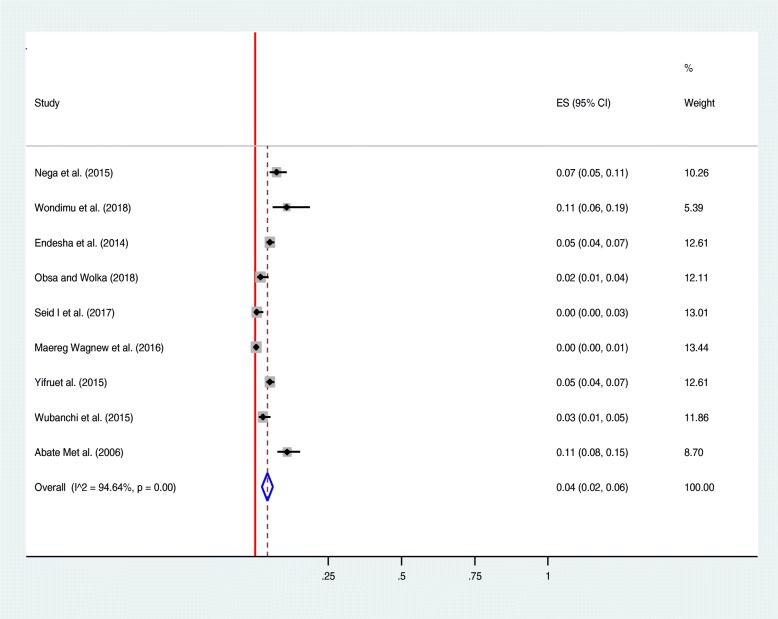
Forest plot displaying rate of Maternal death

**Fig. 3:**
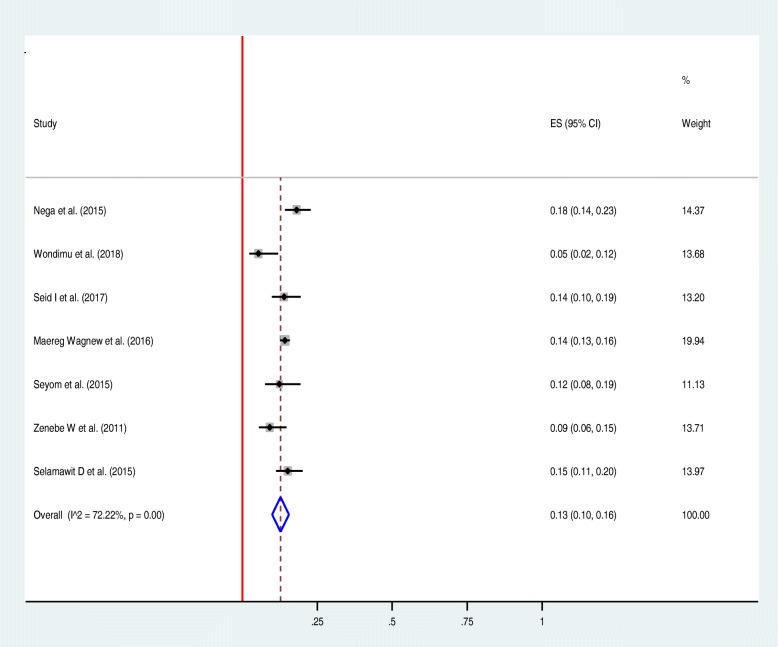
Forest plot displaying rate of HELLP syndrome

Among thirteen studies included in the review, nine studies reported rate of maternal death in women diagnosed to have HDP [20–25, 27, 28, 32]. As illustrated in the forest plot, the overall all rate of maternal death in Ethiopian women with HDP was estimated to be 4% [0.04 (0.02–0.06), I^2^ = 94.64%, *P* < 0.01] (Fig. 2). The test of publication bias using the Egger’s test was non-significant, *p-value* > 0.092 (Fig. 6). Seven studies reported the prevalence of HELLP syndrome [20, 21, 24–26, 29, 31]. As demonstrated in the forest plotthe prevalence of HELLP syndrome was estimated to be 13% [0.13 (0.10–0.16), I^2^ = 72.2%, *p* < 0.01] (Fig. 3). Publication bias was checked by using the Egger’s test that showed non-significant publication bias, *p-value* > 0.428 (Fig. 6). Acute kidney injury was reported by six of the included studies [20, 21, 23, 24, 26, 31]: the maximum rate of acute kidney injury was reported from a study conducted in Jimma specialized hospital (24.2%) [20]; and, the minimum rate was reported from a study conducted in Addis Ababa (6.5%) [24]. Placental abruption complicates as high as 15.3% of women with HDP in Addis Ababa [25]; and, as low as 1.3% in Southern Nations and Nationalities of Ethiopia [30]. The highest rate of pulmonary edema was reported from one study conducted in Jimma specialized hospital (20.1%) [20]; followed by a study from Addis Ababa (17.5%) [25]. Aspiration pneumonia complicates 17.5% of women with the diagnosis of HDP in one study [25].

### Perinatal outcomes of HDP

**Fig. 4:**
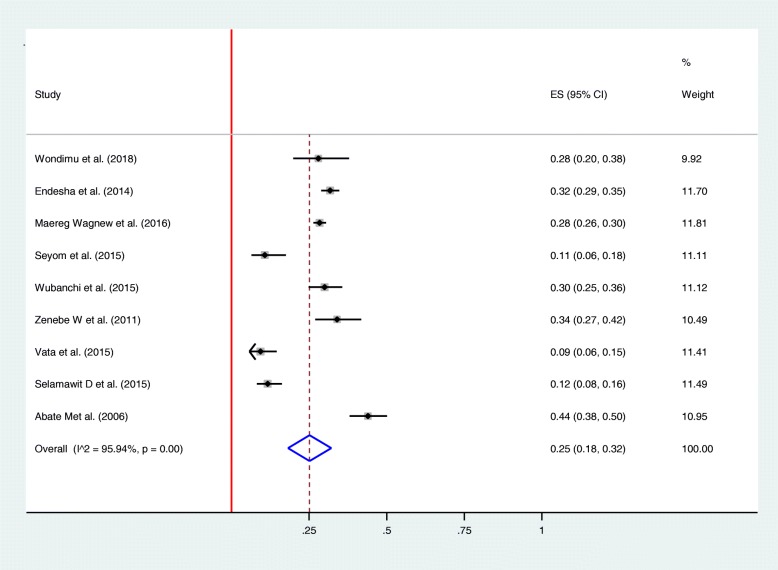
Forest plot demonstrating frequency of Perinatal death

**Fig. 5:**
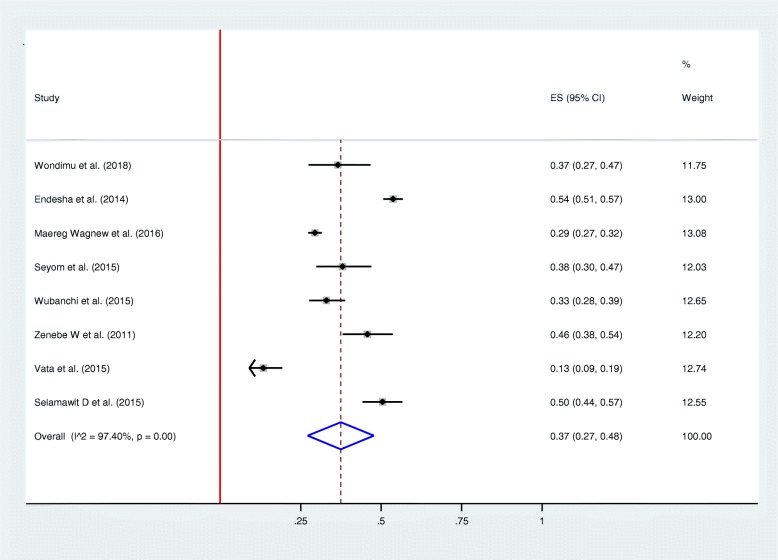
Forest plot showing frequency of Low birth weight

**Fig. 6:**
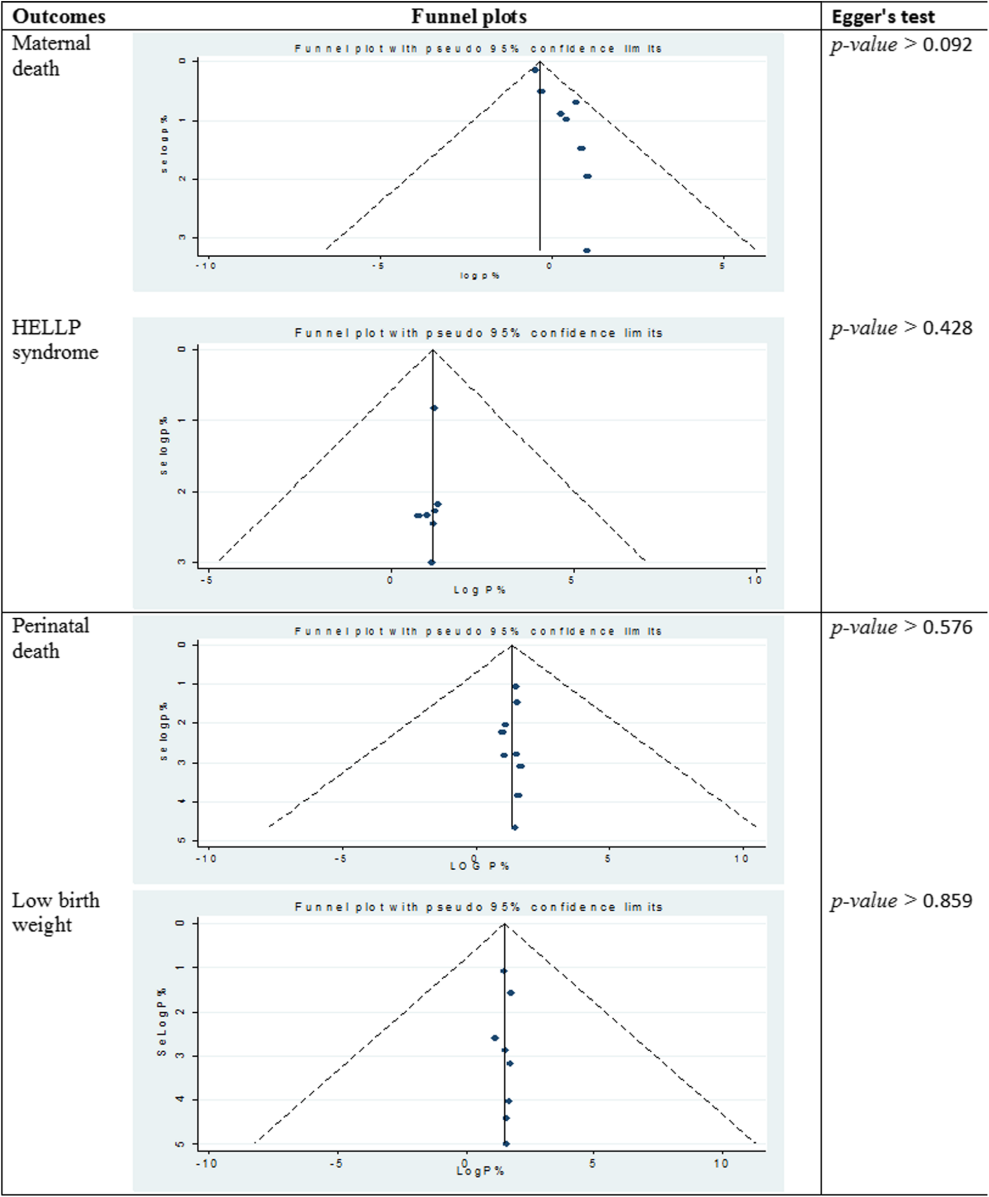
Publication bias of maternal and fetal outcomes

Among thirteen studies included in the review, nine studies reported rate of perinatal death in mothers diagnosed with HDP [21, 22, 25, 26, 28–32]. As illustrated in the forest plot, perinatal death was observed in one-fourth of women [0.25 (0.18–0.32), I^2^ = 95.94%, *p* < 0.01] (Fig. 4). Egger’s test was conducted and illustrated that the publication bias is not statistically significant, *p-value* > 0.576 (Fig. 6). The rate of stillbirths were most common than the rate of early neonatal deaths. For instance, in one study the rate of stillbirth was almost four folds higher than early neonatal deaths (81% vs. 19%) [22]. The frequency of giving birth to a low birth weight neonate was assessed using eight studies [21, 22, 25, 26, 28–31]. The overall rate of having a low birth weight newborn was found to be 37% [0.37 (0.27–0.48%), I^2^ = 97.40%, *p* < 0.01] (Fig. 5). Egger’s test was employed to see for publication bias and found non-significant, *p-value* > 0.859 (Fig. 6). Perinatal asphyxia was reported by six studies [21, 26, 28–31]: the highest rate being 52.8% in Addis Ababa [31] and the lowest rate being 13.4% in Southern Ethiopia [30]. Preterm birth complicates as high as 65.3% of women with HDP in Somalia regional state of Ethiopia [21] to as low as 31% in a study conducted at Oromia regional state [26].

## Discussion

In the current review, 4% of women in Ethiopia diagnosed with hypertensive disorders of pregnancy ended up in maternal death. This finding is similar with a study conducted in Pakistan that reported maternal death rate of 6.23% among women with hypertension [33]. While the case fatality rate for eclampsia ranged from 0 to 1.8% in high-income countries. Such a wide disparity is due to both differences in incidence and quality of obstetric care for hypertensive disease in pregnancy [41]. The rate of maternal death from this review is much higher than a study conducted in Saudi Arabia which reported a maternal death of 1.3%. The Saudi Arabian study was conducted in a tertiary center that may explain the lower reported rate of maternal death as compared to the current finding [34]. This could also be justified by the high number of participants with no antenatal care (ANC) follow up in some of the studies included in the current review. Furthermore, differences in the quality of maternal and neonatal health care service may have been caused this disparity.

In Ethiopia, HELLP syndrome complicates 13% of women with hypertensive disorders of pregnancy. This finding is line with a study conducted by Karumanchi et al. which reported a 10 to 20% rate of HELLP syndrome among women admitted for the diagnosis of preeclampsia [35]. The much higher rate of HELLP syndrome as compared to women without hypertensive disorders (0.5–0.9%) could be explained by the pathological course of the disease. In preeclampsia for example, defective placental vascular remodeling around mid second trimester of pregnancy with the second round of trophoblastic invasion into the deciduas results in inadequate placental perfusion. The hypoxic placenta then releases various placental factors such as soluble vascular endothelial growth factor receptor-1 (sVEGFR-1), which then binds vascular endothelial growth factor (VEGF) and placental growth factor (PGF), causing endothelial cell and placental dysfunction by preventing them from binding endothelial cell receptors. This results in increased platelet activation and aggregation leading to low platelet count, haemolysis and hepatic injury [36, 37].

Pulmonary edema, acute kidney injury, hepatic injury, placental abruption, aspiration pneumonia, and other life treating complications were also reported by included studies. The above mentioned complications were reported by the 2014 world health organization (WHO) multinational analysis using 29 countries from Africa, Asia, Latin America and Middle East [3, 4].

In this review, the prevalence of perinatal mortality among women with hypertensive disorders of pregnancy in Ethiopia was found to be 25%. This finding is in line with another study conducted in Pakistan which reported a perinatal mortality of 17.5% [33]. However, this is in contrary to a Norway study conducted among pregnant women that reported a perinatal mortality of only 9.2%. This disparity may be explained by differences in the quality of follow up a pregnant woman receives [38].

In this review, low birth weight complicates more than one-third of women with HDP in Ethiopia (37%). This rate is much higher than the rates reported in a study conducted in China (6.8%); and, a review conducted among women with chronic hypertension (16.9%). As per the 2016 Ethiopian Demographic and Health Statistics (EDHS) report, the rate of low birth weight in the general population is 13% that could explain the existing higher number of low birth weight in Ethiopia [9].

Perinatal asphyxia, preterm birth and other complications have been also reported in the studies included in the review. The above mentioned complications have been reported by other original articles and systematic reviews [39, 40]. Although the exact mechanisms for the above mentioned perinatal complications are not yet well known, the most acceptable theory for the development of preeclampsia is defective remodeling of spiral arteries. Defective placentation affects utero-placental blood flow and leads to complications such as preterm birth, low birth weight, perinatal asphyxia, and fetal growth restriction [14, 15]. An Indian study reported that the most common neonatal complication was prematurity (23.65%), low birth weight (7.52%) and intrauterine growth restriction (9.67%) [16].

### Strength and limitations of the study

This review is the first to review the maternal and fetal outcomes among women with all types of HDPs in Ethiopia. The limited numbers of studies evaluating outcomes made it impossible to generate strong conclusions. The included studies were also conducted primarily in tertiary health centers, and the data may not be representative of outcomes in low-level facilities or in the community. Furthermore, there is a high level of heterogeneity among the included studies that should be taken in consideration while using the results of the review. Despite these possible limitations, the review provides useful information that may contribute to both the filling of the gaps in the national maternal morbidity research agenda and guiding practice and policy about the most frequent complications of HDP.

### Conclusions and recommendations

This review demonstrated the high prevalence of perinatal and maternal mortality among pregnant women with one of the HDP in Ethiopia. Moreover, other severe maternal and perinatal complications such as HELLP syndrome, placental abruption, pulmonary edema, hemolytic anemia, renal damage, prematurity, perinatal asphyxia, as well as low birth weight were also commonly reported.

Based on the above mentioned results, it is recommended to intervene at three levels so as to improve the maternal and fetal outcomes of hypertensive disorders in Ethiopia and to ensure the safety of a pregnant woman and her baby. Giving the above mentioned fact of poor antenatal care (ANC) service utilization, community based health education can be one possible level of intervention to improve the health outcomes of women with HDP by improving maternal health service utilization. The second possible level of intervention could be early detection and early referral of pregnant women with hypertensive disorder. Policies and strategies that may enhance the health professionals’ capacity should be advocated. The other possible level of intervention could be, optimizing the quality of care that a pregnant woman with hypertensive disorders receives. Additionally, it is recommended to have easily available and affordable laboratory testing; frequent maternal and fetal monitoring; increase accessibility of antihypertensive and anticonvulsive drugs; increase preparation for neonatal resuscitation; and, improving quality of neonatal intensive care units. It is also recommended to develop a uniform systematic registration system of maternal and perinatal outcomes of HDP in the country.

## Data Availability

All relevant materials and data supporting the findings of this review are included within the manuscript.

## Abbreviations

ANC: Antenatal care
CI: Confidence interval
HDP: Hypertensive disorders of pregnancy
WHO: World Health Organization
